# Assessing road criticality and loss of healthcare accessibility during floods: the case of Cyclone Idai, Mozambique 2019

**DOI:** 10.1186/s12942-022-00315-2

**Published:** 2022-10-12

**Authors:** Sami Petricola, Marcel Reinmuth, Sven Lautenbach, Charles Hatfield, Alexander Zipf

**Affiliations:** 1 GIScience department, Institute of Geography, Heidelberg University, Heidelberg, Germany; 2 Heidelberg Institute for Geoinformation Technology gGmbH (HeiGIT), at Heidelberg University, Heidelberg, Germany

**Keywords:** Disaster, Public health, Routing, OpenStreetMap, Accessibility, Network

## Abstract

**Background:**

The ability of disaster response, preparedness, and mitigation efforts to assess the loss of physical accessibility to health facilities and to identify impacted populations is key in reducing the humanitarian consequences of disasters. Recent studies use either network- or raster-based approaches to measure accessibility in respect to travel time. Our analysis compares a raster- and a network- based approach that both build on open data with respect to their ability to assess the loss of accessibility due to a severe flood event. As our analysis uses open access data, the approach should be transferable to other flood-prone sites to support decision-makers in the preparation of disaster mitigation and preparedness plans.

**Methods:**

Our study is based on the flood events following Cyclone Idai in Mozambique in 2019 and uses both raster- and network-based approaches to compare accessibility to health sites under normal conditions to the aftermath of the cyclone to assess the loss of accessibility. Part of the assessment is a modified centrality indicator, which identifies the specific use of the road network for the population to reach health facilities.

**Results:**

Results for the raster- and the network-based approaches differed by about 300,000 inhabitants (~ 800,000 to ~ 500,000) losing accessibility to healthcare sites. The discrepancy was related to the incomplete mapping of road networks and affected the network-based approach to a higher degree. The modified centrality indicator allowed us to identify road segments that were most likely to suffer from flooding and to highlight potential backup roads in disaster settings.

**Conclusions:**

The different results obtained between the raster- and network-based methods indicate the importance of data quality assessments in addition to accessibility assessments as well as the importance of fostering mapping campaigns in large parts of the Global South. Data quality is therefore a key parameter when deciding which method is best suited for local conditions. Another important aspect is the required spatial resolution of the results. Identification of critical segments of the road network provides essential information to prepare for potential disasters.

**Supplementary Information:**

The online version contains supplementary material available at 10.1186/s12942-022-00315-2.

## Background

Induced by climate change and human activities, the frequency and intensity of extreme natural disasters are growing continuously [1]. According to the UN, changes in weather and climate account for 90% of disasters and the number of disasters including floods, droughts, heatwaves, and devastating storms has doubled since the’80 s [2]. While natural disasters are occurring in most regions of the globe [3], their impact remains dependent on the local context as low-income countries have fewer resources to prepare, mitigate, and recover [4]. Cyclone Idai which hit the southeastern coast of Africa in March 2019 is an example of a large-scale event affecting low-income countries.

Beyond the direct damages caused by natural disasters, the consequences often depend on the cascading effects induced. This results from a series of failures of interconnected or dependent infrastructure causing “physical, social or economic disruption”: “cascading effects are associated more with the magnitude of vulnerabilities than with that of hazards” [5]. In some regions of the Global South, such cascading effects can cause tremendous damage in addition to direct physical effects on populations. Access to essential services can swiftly drop due to the lack of backup roads within the network, poor quality of building materials of health facilities, and the remoteness of populations [6]. The loss of accessibility to health facilities trigger, in turn, subsequent risks like epidemics [7], delayed treatment, and jeopardize health prevention and promotion programs. For example, outbreaks of vaccine-preventable diseases, such as diphtheria or measles in Bangladesh and the Central African Republic respectively, emerged when populations lost access to healthcare [8].

Rapid deployment of emergency responses is key to reducing the acute effects of disasters on communities. Therefore, it is necessary to develop swiftly calculable and easily interpretable metrics based on open data that can be used to identify the most affected populations, particularly in the Global South where international actors often lack knowledge of the local context and the availability of key official information is scarce [9]. In the preparedness, mitigation, and recovery phases of the disaster management cycle, the focus switches toward the operationality of the infrastructures providing access to healthcare. The concept of infrastructure criticality is commonly understood in literature and disaster management [10, 11] as the propensity of an infrastructure to affect the network’s connectivity in case of failure [12]. Thus, assessing the lack of resilience of one or several road segments which may inhibit access to health facilities allows to identify the potential weak points in the road network and gives actionable data options for disaster management [13, 14]. 

### State of the art and research gaps

In the last decade, many metrics have been developed [15] to measure the potential access of populations to healthcare as defined by Guagliardo [16]. Recent metrics such as gravity-based and floating catchment area measures [17] offer advantages, but studies [15, 18], have shown that the complexity of interpretation, implementation, and the need of detailed data (facility type and capacity) are barriers to their use by practitioners in real-life scenarios. In contexts of the Global South where data is often scarce or not available and for emergency response applications, simpler metrics which require less detailed data are often prefered. For instance, raster-based approaches have been used at a global scale [19], for the analysis of individual disasters [20], and for the optimization of road development in rural Nepal [21]. Other studies have investigated the potential access to health facilities based on network-based methods at the country level [22]. Examples involve the analysis of the impacts of conflicts [23], epidemics [24], and flooding [13, 25, 26]. The two approaches build on two fundamentally different representations of space. For raster-based approaches, travel time per cell is usually calculated as a function of street presence and street characteristics, land use, and terrain. In contrast, network-based approaches model travel time primarily based on the road network and its properties. Previous studies have compared the two approaches. However, they did not conclude on the best approach but rather highlighted some differences to be adapted according to the context [27, 28]. Our study aimed at a more detailed comparison of the two approaches for the assessment of the share of the population vulnerable to a loss of accessibility to healthcare in the specific context of emergency response during a flood disaster with datasets accessible in a timely manner.

One important aspect of disaster preparedness is the criticality of infrastructure. In our case the focus is on the criticality of the road network. This involves the question of how sensitive accessibility to healthcare sites is to the failure of individual road segments by a disaster. Studies using graph theory in an urban context [29–31] have shown the relevance of centrality indicators for this purpose. Other studies used this indicator to assess the vulnerability of a road network in the context of floods at the urban level [25, 32] as well as at the national level [33]. Betweenness centrality of a network node—or edge—[34] sums the number of shortest paths between all pairs of nodes of the network to go through this node. A higher betweenness centrality score of a node—or edge—would imply a higher level of importance within the network and thus, potentially a lower availability of alternative roads in case of a cut-off. However, betweenness centrality has been criticized as overly simplistic [35] and extensions of the approach have been suggested to consider origin–destination distribution [36] or node importance within the network [37]. One important aspect that has so far not been adequately considered is the edge betweenness centrality with respect to critical infrastructure and population distribution. Most studies consider road networks in their globality and do not differentiate with respect to the usage or specific destination. In most cases, important facilities such as health centers and hospitals are not homogeneously positioned within the network and a low criticality assessment may hide some specific usage disparities if, for instance, the health facility is in an isolated part of the network. Moreover, betweenness centrality is directly connected to the network topology and the density of its nodes. Thus, if the road network is not completely mapped or if the repartition of nodes is not correlated with the real network (i.e.: density of nodes differs from density of population nucleus), the betweenness centrality score will be biased. Furthermore, the analysis of betweenness centrality remains limited due to its high computational performance requirements [38]. A focus on targeted usage of the network would reduce the computational requirements and thus allow a more extensive area of analysis. To overcome those issues, we modified the edge betweenness centrality analysis to propose and test a road criticality indicator.

As geodata for most parts of the global South is sparse, one has to consider the completeness of the road network data as an important factor of uncertainty. OpenStreetMap (OSM), as an open source geodataset provided by volunteers, is often the best available data source for many regions and is an important data source for the analysis of accessibility and the characteristics of road networks. However, the quality of OSM, as with most volunteered geographic information (VGI), is spatially heterogeneous [39, 40]. Therefore, it is necessary to assess data quality for the case study region. In the absence of external reference data of a higher quality, one has to rely on intrinsic data quality assessments. The different aspects of data quality are thereby estimated by an analysis of the history of OpenStreetMap [41–43]. Moreover, there is generally an unquantifiable time offset between changes of objects in reality and the survey of these changes into OSM.

We selected the case study of Cyclone Idai as a severe event in terms of the number of people affected, the area devastated, and the magnitude of international response. The aims of our study are twofold:

1. Identifying the most appropriate method to assess potential access to healthcare and to identify vulnerable populations in the aftermath of a natural disaster based on open data.
2. Assessment of a modified version of betweenness centrality indicator to assess road infrastructure criticality and support preparedness and mitigation plans of disaster-related loss of healthcare accessibility.

### Case study: Cyclone Idai

**Fig. 1:**
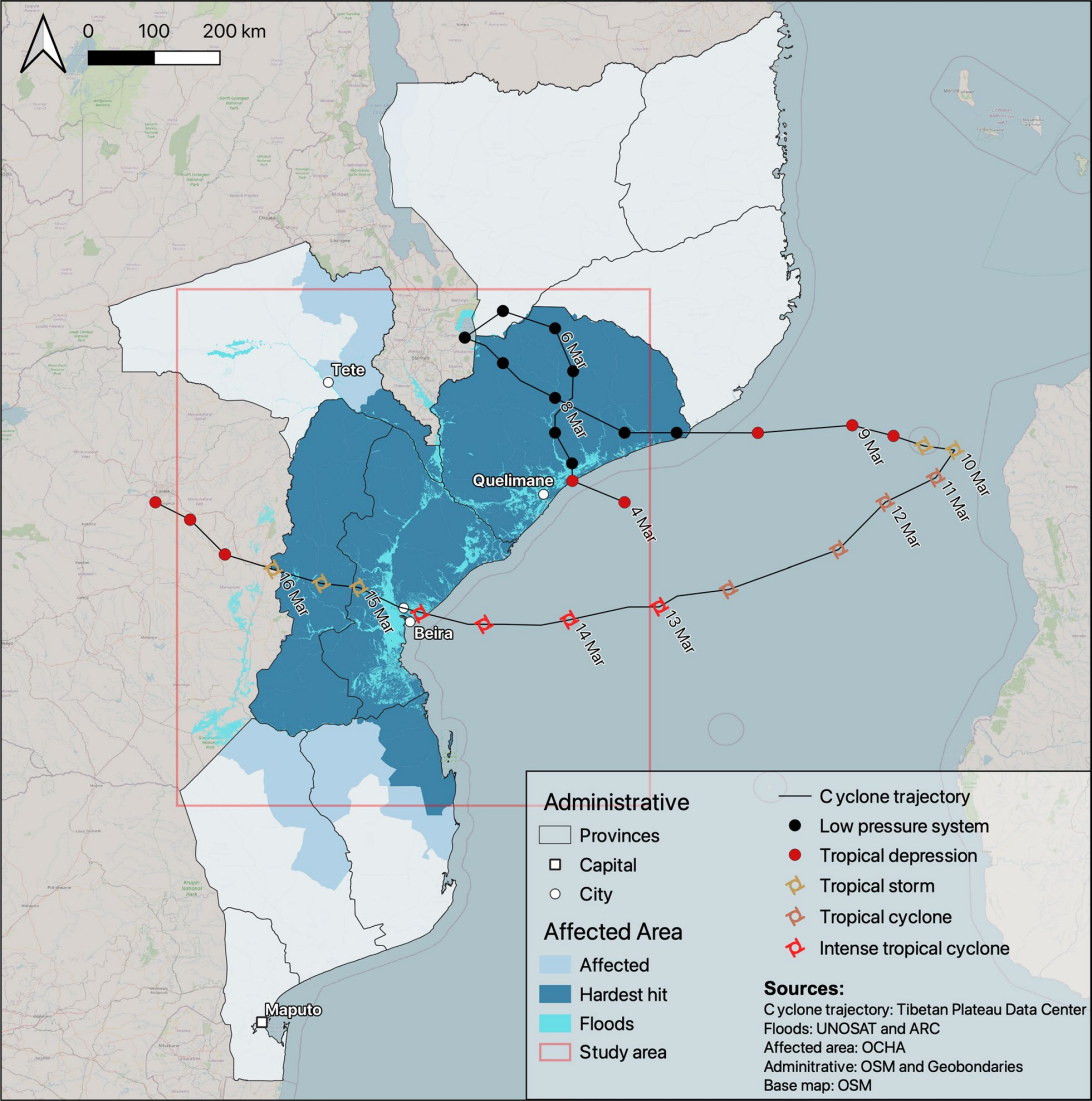
Trajectory and areas affected by Cyclone Idai over Mozambique in March 2019 and the study area

The UN's World Meteorological Organization declared Cyclone Idai as “one of the worst weather-related disasters in the southern hemisphere” [44]. The cyclone impacted the southeastern coast of Africa in March 2019 (Fig. 1). Between the 4th and 21st of March, it made landfall twice causing tremendous damage in Mozambique, Malawi, and Zimbabwe, affecting over three million people [45]. Furthermore, at least 45 healthcare centers were partially or totally destroyed in Mozambique [46].

During the first days after the passing of Cyclone Idai, hundreds of humanitarian organizations and international agencies activated their emergency response systems to provide first aid to affected populations and to prevent epidemic outbreaks, such as cholera. The needs assessments and the effective division of relief efforts by actors was challenged by the lack of contextual knowledge of the area as most of intervening organizations did not have a permanent presence prior to the event [47, 48].

## Methods and data

### Data availability and completeness

To compare different methods, we used open access data on the road network and health facilities, demographic, and administrative data as well as remote sensing-based flood masks. For the raster-based accessibility analysis, a friction surface representing the travel time per cell of a tessellation was provided by the Malaria Atlas Project [49]. It was calculated as a function of street presence and street characteristics, land use, and terrain.

The flood extents were provided by two different datasets which focused on complementary areas. Both were combined in a vector layer and used as the flood mask for the following analysis steps. To lower the computational burden, the geometries were simplified using the Visvalingam algorithm [50] with only one percent of points retained. The first flood extent dataset was based on results published by the World Food Program [51] on the 21st of March, four days after the end of the cyclone. The flood extent was retrieved from a 22 km resolution microwave radiation satellite imagery from SSM/I, AMSR2, and GMI sensors and downscaled with a DEM to a 90 m resolution raster dataset [52]. The images were acquired on a daily basis between the 12th and the 20th of March 2019 and aggregate the extent of the detected floods. The second dataset was published in vector format on the 26th of March 2019 by UNOSAT [53] and was acquired by the Sentinel-1 radar sensor with a resolution of 10 m. The product presents the cumulative floods detected on the 13th, 14th, 19th, 20th, and 26th of March. The whole analysis was restricted to the extent of Mozambique, using the administrative boundary from geoboundaries.org [54].

To assess quantitatively the vulnerabilities of the population regarding healthcare accessibility, we extracted population counts from a raster dataset by worldpop [55]. Worldpop [56] provides several datasets of gridded population counts with a 100 m resolution. We used the 2020 constrained top-down method adjusted with UN population estimates for Mozambique.

Road network and health facility data were obtained from OpenStreetMap. As the completeness of the dataset is dependent on volunteer contributions and increases over time, we used the latest extract available at the moment of the analysis in April 2021. The road network was extracted from Geofabrik.de [57]. For the analysis of the flood event, we used the tool Osmosis [58] to simulate the road network alteration due to the flood. All nodes of the road network that were intersecting with the flood mask were removed. The healthcare facilities were obtained via the ohsome API [59]. We considered 1059 healthcare facilities recorded in OpenStreetMap for Mozambique, including any OSM object that bears a healthcare function no matter the level, such as primary, secondary and tertiary facilities.

The acquisition of the OSM dataset at the time of the analysis instead of the time of the event represents a potential bias. To assess this bias and verify the data quality, we performed an intrinsic data quality approach. We accessed the OSM history to assess the evolution of the dataset over time on relevant features. We used Ohsome API [59] to filter the contributions on specific highways (*highways* = *motorway, trunk, primary, secondary, tertiary, unclassified, track, path)* and all health facilities that can provide primary health care (*amenity* = *clinic, health_post, doctors, healthcare* = *doctors, clinic, midwife, nurse, center*) including also hospitals (*amenity* = *hospital, healthcare* = *hospital, building* = *hospital*). Additionally, we analyzed the evolution of user activities on these features. A sudden increase in the mapped features in the flooded area in the wake of the cyclone would give an insight into the lack of data completeness at this time and the activation of remote mapping campaigns to address it. On the contrary, a sudden decrease in user activities would illustrate a drop in the interest in mapping the area (Fig. 1).

**Fig. 2:**
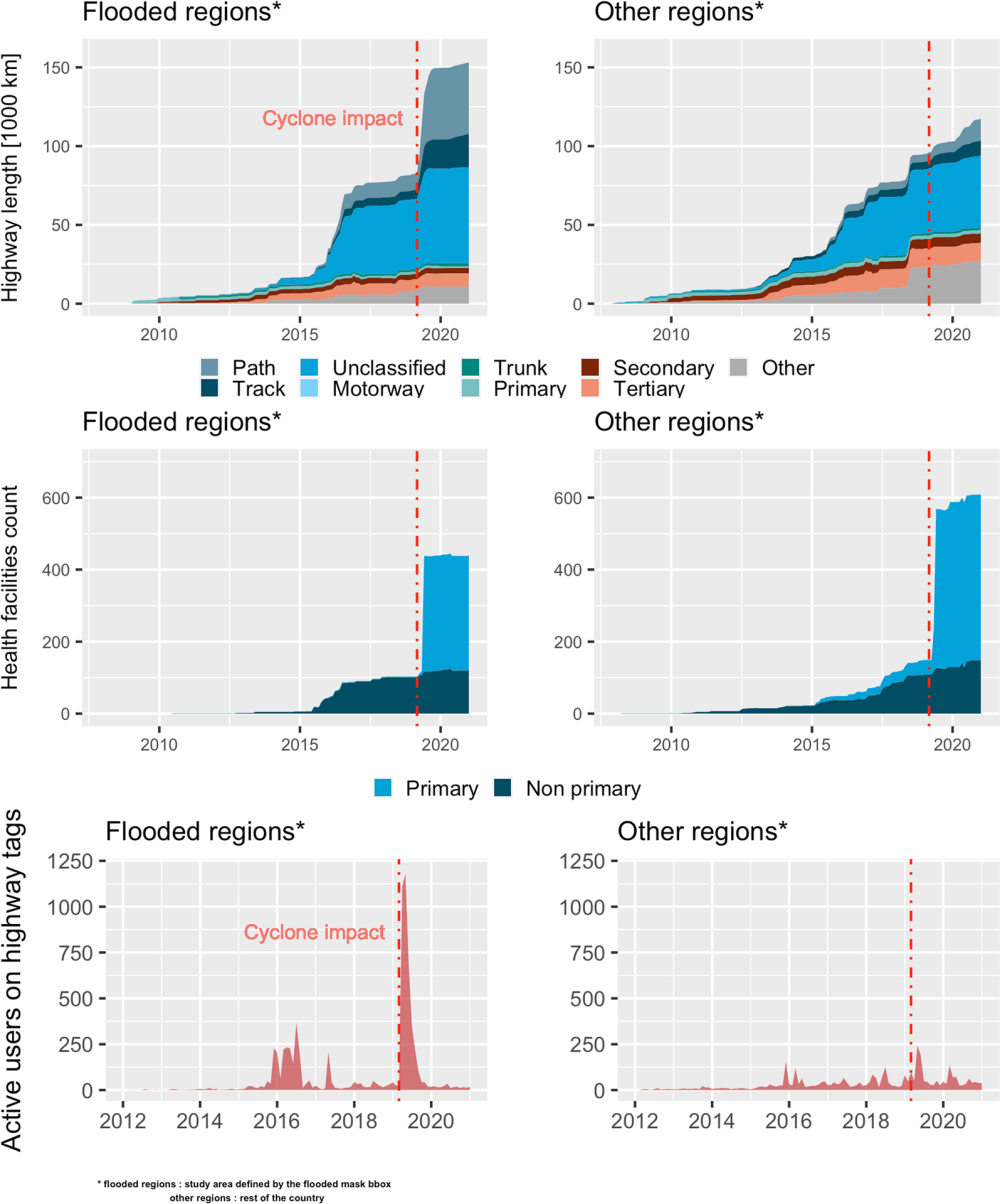
History analysis of OSM contribution: number of objects and active users over time. Flooded regions represent the study area, other regions represent the rest of the country. The dashed line marks the impact date of the cyclone in March 2019

After the disaster, the total length of mapped roads increased substantially in the flooded regions (Fig. 2). At the time the cyclone hit, around 80,000 km of roads were mapped, while mapped road length increased by a factor of 1.9 to almost 150,000 km in the following months. For the rest of the country the increase was much lower (from 90,000 km to 105,000 km, an increase by a factor of 1.2). Regarding health facilities, few primary facilities were present in the OSM database prior to the disaster while several hundred were added in the following weeks all over the country. The history of OSM user activity showed a peak followed by a quick drop-off during the humanitarian response in April 2019. We interpret this as a fading of interest and the end of the OSM community mobilization. Therefore, the contributions plateauing in the flooded regions could either result from the completeness of the database or from the end of mapping activities triggered by the disaster.

In Additional file 1, we present more in depth data completeness assessment, including a proxy measure based on external dataset and the field observations from a humanitarian field worker.

### Loss of healthcare accessibility analysis

Our analysis estimated the potential access to health facilities, the loss of accessibility and the impacted population by a loss of accessibility. The potential access to healthcare analysis was run independently in both flooded and normal conditions. Both were then compared based on their geometrical difference (see Additional file 2: Fig. S3 for detailed workflows). Thereby, we identified the areas which experienced a loss of accessibility to health facilities. Using the worldpop dataset, we finally estimated the number of inhabitants by access time under both conditions and in the areas experiencing a loss of accessibility. The results allowed us to measure the impact of the floods on the accessibility of the population to health facilities.

The healthcare accessibility analysis was done with both network- and raster-based methods and their results were compared to identify the most appropriate approach depending on the contextual characteristics. The literature on healthcare accessibility tends to focus on a transportation mode based on motorized vehicles. Nevertheless, in many Sub-Saharan countries, including Mozambique [60], walking remains the main means of transport especially in disaster situations and in rural areas. Therefore, we considered both transportation profiles.

The estimation of accessibility with a network-based approach was based on isochrones [61] which represent the area accessible from one point within a given time. The isochrones were calculated for the 1059 health facilities in Mozambique using the openrouteservice API [62]. Openrouteservice is a free of cost routing engine available as open source software which can also be used as a service via openrouteservice.org. It uses OSM road network data to build a weighted graph according to road categories, speed limits, and traffic restrictions. Hence, the calculation was restricted to road segments and did not consider open space routing for areas not connected by the road network. We calculated isochrones based on the time required to reach the nearest health facility. We used 10 min intervals capped to a maximum of one hour for the driving-only profile. For the walking-only profile, we also considered 10 min intervals which were then aggregated by hour to a maximum of six hours. As the loss of accessibility calculations rely on the difference in travel times and the geometric differences between the respective isochrone polygons, it resulted in potential precision error. This potential error was equal to the time interval (10 min).

The raster-based method calculates a least-cost path on every pixel of the raster grid to access the same healthcare facilities. The algorithm is based on a friction layer that attributes a time cost to cross a pixel. We used a walking-only friction layer as well as a friction layer combining walking with motorized transportation where road and train information is available. The friction layer integrated data on land cover, therefore the calculation was not restricted to the road network and considered travel on any surface with an adjusted speed. The gDistance (see Additional file 2: Table S1) R package was used to compute the least-cost path. In this approach, we altered the cost of the flooded areas to *NA* values to forbid travel. The continuous result was categorized into time intervals (10 min) to allow a comparison with the network-based approach.

###  Road criticality analysis

The analysis of the road criticality requires a graph representation of the network. We used openrouteservice [62] to export the weighted graph. To measure road criticality we developed a modified version of the edge betweenness centrality [63], the targeted edge betweenness centrality (TEBC) indicator. Instead of considering the shortest paths between all possible pairs of nodes in the network (as for edge betweenness centrality), the TEBC calculates the road segment usage frequency among the shortest paths between targeted destination points (in our application healthcare facilities) and departure points of the area of interest (Eq. 1). The departure points were set as population nuclei and were computed from the centroid of the cells of the worldpop dataset after aggregation to a 1 km grid. For each population nucleus, the targeted destination points were selected within a buffer zone of 20 km. This filter implied the assumption that a person has an uniform probability to visit any of the destination points within the buffer zone. To avoid any bias due to the heterogeneity of the destination density, the frequency was weighted by the number of destinations accessible from each departure point.

1 $$TEBC\left(i\right)=\sum_{s,{ d}_{s}}\frac{{g}_{s{ d}_{s}}^{i}}{{N}_{{ d}_{s}}}$$

where $i$ is the specified segment of the network, $s$ is one of the departure points, ${d}_{s}$ is the destination points within the range of $s$, ${g}_{s{d}_{s}}^{i}$ is equal to 1 if the shortest paths between $s$ and ${d}_{s}$ passes through $i$ and 0 otherwise, ${N}_{{d}_{s}}$ is the number of destinations ${d}_{s}$ within the range of $s$. The calculation of shortest paths was done with the R package sfnetworks.

Segments with higher TEBC scores are more frequently crossed. A similar TEBC score for all segments indicates a lower overall road criticality level within the network as there are many alternatives to any road disruptions. On the contrary, a wider range of TEBC scores indicates a stronger vulnerability of the network as the loss of one main axis could isolate parts of the network.

The TEBC was calculated for the road network for both normal and flooded conditions. For the flooded case, road segments inside the flood masks were not considered. The scores for both cases were then compared. For a single road segment, a decrease in the TEBC score after flooding indicates higher criticality as it is not able to provide access due to the floods. In a network with a *hub-and-spoke* topology [64], these criticalities could result in a lack of resilience if no mitigation action is taken, such as flood walls. On the other hand, an increase in the score would identify the segment as an important backup road. Such roads should get special attention in disaster preparedness plans as they have major importance in disasters.

### Software and data

The data processing was done with R 4.0.5 [65]. The scripts and documentation are available in open access. In addition, the complete list of used R packages is available in the Additional file 2: Table S1. Osmosis was used to remove the OpenStreetMap data within the extent of the floods. Openrouteservice was installed locally using Docker [66]. The maps were created with QGIS 3.18 [67].

## Results

### Loss of healthcare accessibility analysis

**Fig. 3:**
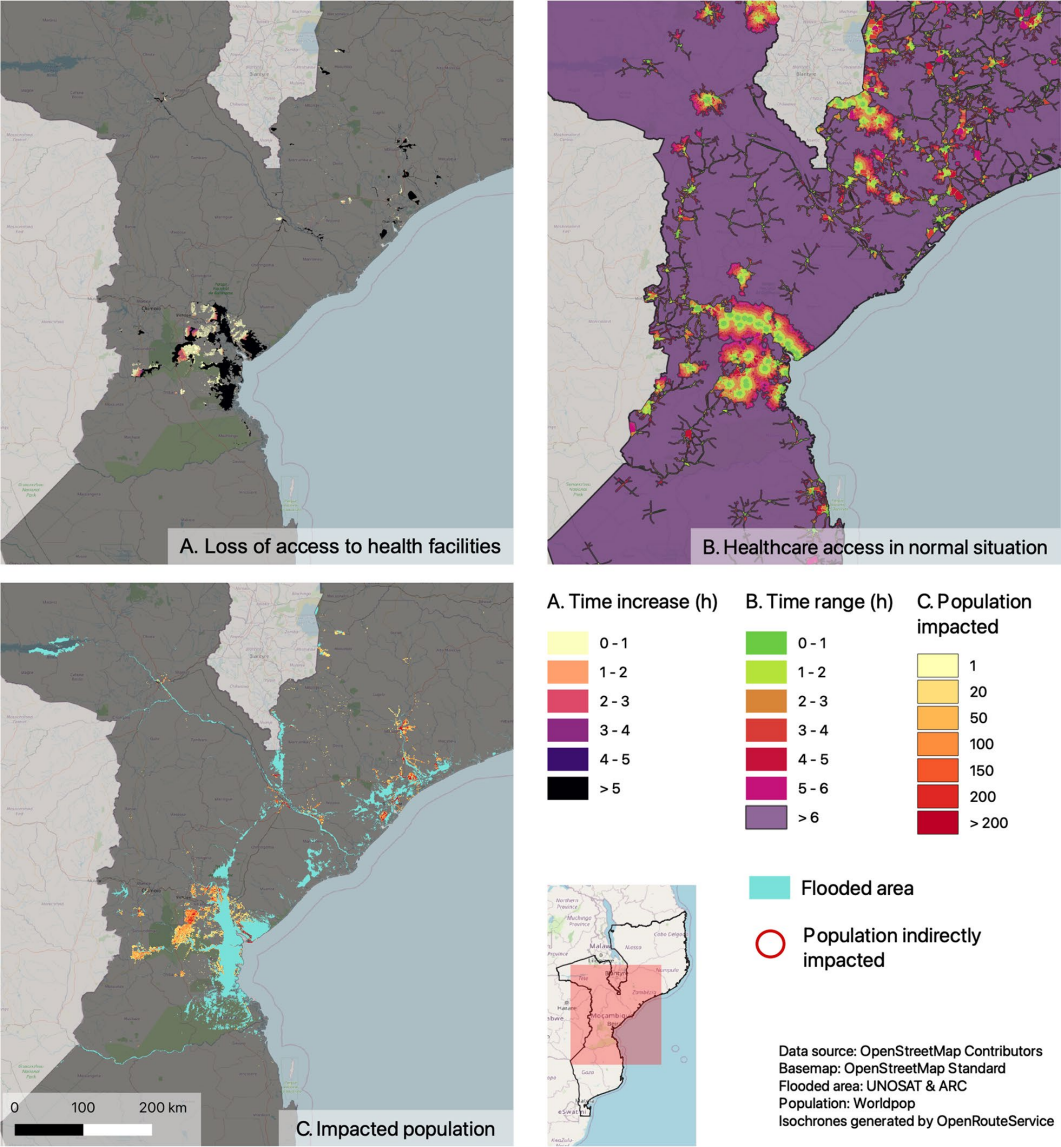
Analysis results for the network-based method and walking transport mode. **A** Loss of accessibility as measured by the increase in travel time between the normal conditions and after flooding. **B** The absolute access time to the closest facility in normal conditions. **C** The population nuclei impacted

With the analysis of potential access to healthcare facilities, we observed the localization of all the populations affected by the floods. We were able to identify the population nuclei affected, even though they were not in the direct vicinity of the floods (Fig. 3).

The results of the raster-based and network-based methods differed remarkably with respect to the estimated potential access time (Fig. 5). For the walking-only profile the raster-based and the network-based approach estimated 3.01 million (9.61% of the country population) and 14.76 million inhabitants (47.23% of the country population) respectively that lived beyond walking distance (more than six-hours walking) of the nearest health facility under non-flooded conditions. The results were similar for the car profile with 4.52 millions inhabitants (14.46% of the country population) living beyond driving distance (more than 1-h driving) estimated by the raster-based approach and 14.15 millions inhabitants (45.29% of the country population) estimated by the network-based approach.

**Fig. 4:**
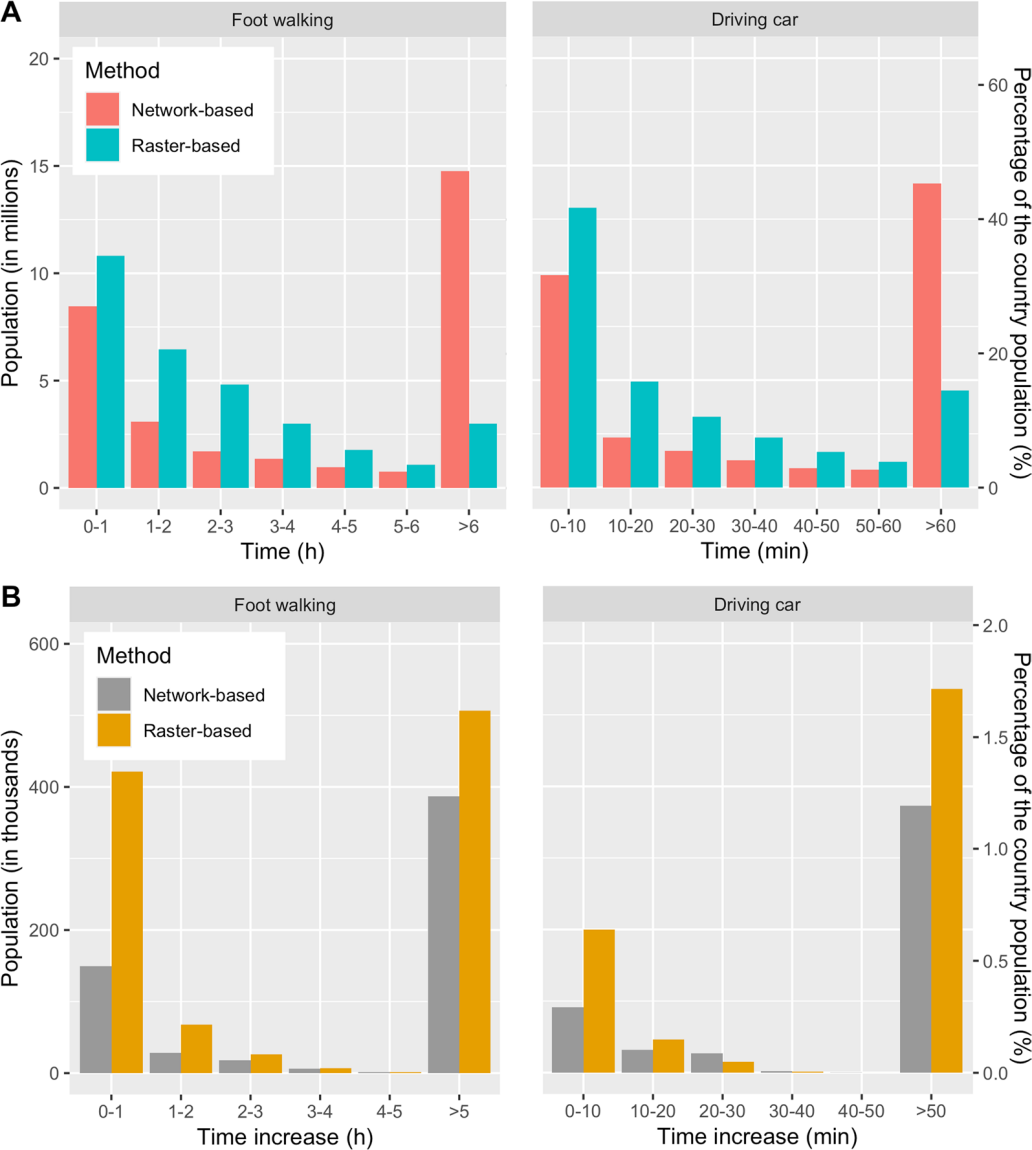
Comparison of raster- and network-based methods for both walking and driving profiles. **A** Estimate of access time to healthcare facilities during normal conditions. **B** Estimation of loss of accessibility to healthcare facilities due to flooding caused by Cyclone Idai

Considering the loss of potential access by estimating the increase in travel time to reach the closest healthcare facility after the floods, the two methods also differed by a significant degree (Fig. 4). According to the raster-based approach, a total of 1.03 million (3.29%) inhabitants experienced an increase in walking time compared to 0.59 million (1.88%) according to the network-based method. Moreover, the distribution of the population experiencing a loss of accessibility by time range differs between the methods. The raster-based method estimated that half (50.8%) of affected inhabitants experienced an increase in walking time less than five walking hours while the network-based method estimated that the majority (65.59%) of inhabitants experienced a more dramatic increase in walking time (more than five hours). When considering motorized transportation, the results of the analysis were similar with an estimated total of nearly 0.8 million (2.56%) inhabitants experiencing an increase in driving time with the raster-based approach and close to 0.53 million (1.68%) inhabitants with the network-based approach. Both approaches estimated that the majority of the affected population experienced an increase in driving time of more than 50 min (67.2% for the raster-based approach and 71% for the network-based approach).

We compared the size of the areas (see Additional file 2: Fig. S4) within a six-hour walking range to healthcare facilities for both the raster- and the network-based approaches. It covered respectively 8.8% (69,164 km^2^) of the country surface for the network-based approach and 64.6% (507,516 km^2^) for the raster-based approach. As expected, the shape of the network-based accessibility closely followed the presence of the road network, while the raster-based accessibility assembled a buffer-like shape around health facilities with a much wider extent. The network-based approach represented accessibility at a much finer spatial resolution in areas with a higher road network density such as the urban areas of Dondo and Beira in Sofala province (see Additional file 2: Fig. S5).

### Road criticality analysis

**Fig. 5:**
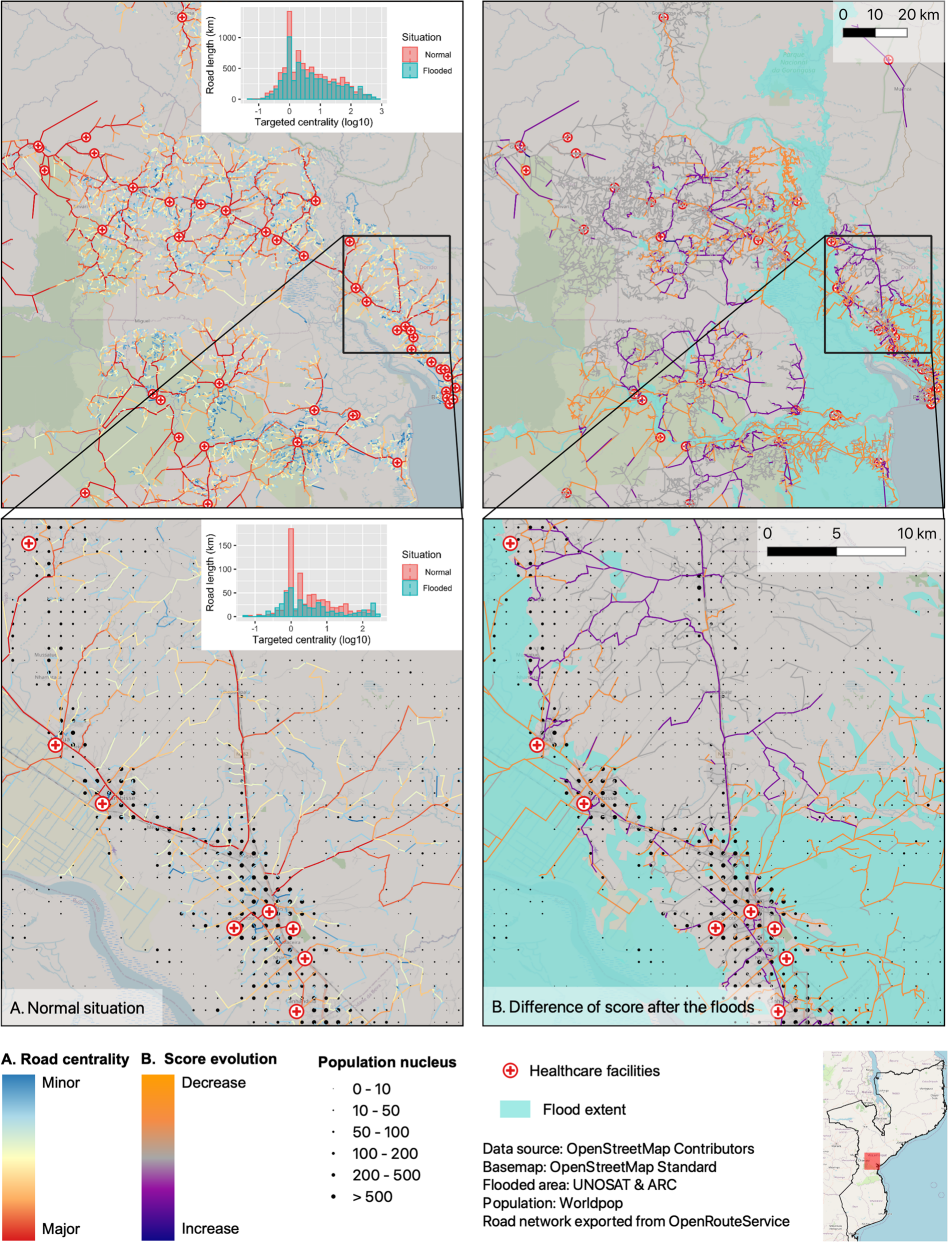
Road network analysis for the driving profiles. **A** Normal conditions before the flood event. **B** Evolution of scores after the floods induced by Cyclone Idai. The lower row shows a close-up of the area surrounding the city of Dondo

In the focus area that we investigated, the flood reduced the number of usable road segments (Fig. 5). The targeted centrality score decreased due to flooding for most road segments but a few increased in centrality. This demonstrates the importance of backup routes for accessing health facilities.

While some of the road segments had high centrality scores both for normal and flooded conditions, other major roads experienced a reduction in their centrality scores or even a reduction to zero if flooded. Access to health facilities should avoid these road segments. If drivers follow previously known routes this would lead to a further reduction in accessibility. A select number of road segments with low or medium centrality scores during normal conditions increased in targeted centrality after the flood event. These road segments could act as backup roads in the case of a flood event if the main roads are not operational. If they were used after such a flood event they could help to avoid a total loss of accessibility for parts of the population even though access time to health facilities would increase considerably.

## Discussion

### Data availability and completeness

In general, we recommend performing an intrinsic data quality assessment for OSM based data since the accessibility results and the targeted centrality analysis depend on the quality of the health facility locations. For the network approach the quality of the road network is also highly relevant. We acquired OSM data from April–May 2021, two years after the disaster. Therefore, the OSM data might be partly inaccurate. However, we can assume that the additionally mapped features represent features that existed already some years ago as the intrinsic data quality assessment revealed that the region was only incompletely mapped. Especially as the remote mapping campaigns launched after the event led to a significant increase in mapped features which are unlikely to have been constructed after the event.

The use of Ohsome Quality Analyst (see Additional file 1) indicators such as the comparison of OSM features and population density can provide an approximation of the completeness of the OSM dataset. Nevertheless, we were only able to compare buildings and population densities. The use of indicators comparing road length density and population density would be better proxies in the future to assess the completeness of the road network. The use of such indicators could then support the decision on the most suitable accessibility method. The results of the OSM data quality and completeness confirmed the choice of using recent data as the available data at the time of the cyclone would have been inadequate for ensuring relevant accessibility and road criticality analysis.

After a disaster event, many infrastructure elements might have been damaged or destroyed. While it is possible to map such damage in OSM, this is not frequently done. This might have led to an optimistic estimation of accessibility to healthcare facilities. We therefore strongly recommend to use field observations for cross-validation where possible. For our case study, an exhaustive and reliable list of damaged health facilities was not available. We assumed that all facilities remained fully operational after the disaster which, is a known limitation of the workflow as shown by the observation of the humanitarian field worker (see Additional file 1: Fig. S2) and the post-disaster assessment report [68].

As identified in the OSM data completeness analysis, the healthcare facilities list within OSM might not be complete as well as the classification accuracy of healthcare facilities cannot be guaranteed. If such data issues are indicated by an intrinsic data quality assessment, it might be worth considering the use of complementary datasets such as the list of health facilities in Sub-Saharan Africa published by the World Health Organization [69] or a combination of several datasets as provided by Afrimapr [70].

### Loss of healthcare accessibility analysis

According to OCHA [45], Cyclone Idai affected around 1.85 million people. Nevertheless, Cyclone Idai had variable impacts across these populations: loss of shelter, increased food insecurity, increased need of WASH services, and loss of accessibility to healthcare, but no disaggregated figures were published. Our network-based and raster-based analysis, respectively, found an estimated 0.59 million and 1.03 million persons experiencing a loss of accessibility to health facilities. Taking into account the limitations of our methods, discussed below, these results are coherent with official numbers.

Our analysis indicated some limitations for both the network-based and the raster-based approaches, which should be considered when selecting an approach for different use cases (see Table 1).

**Table 1:** Comparison of network- and raster-based methods with open data

	Network-based	Raster-based
Critical datasets	OSM network	Friction layer
Dataset update frequency	**Daily** The scope of a daily dataset update is not known as it is based on VGI	**No info available** Last update: 2020
Data incompleteness exposure	**Very high** The calculation is only based on the road network, thus the analysis is limited by the completeness of the mapped network	**Medium** Friction layer construction mitigates the lack of road network completeness
Spatial resolution	**100 m** Area extent is a vector geometry and thus does not have resolution limitations. Population datasets were available at a 100 m resolution	**1 km** Friction layer was available at a 1 km resolution
Time resolution	**Time intervals** The loss calculation cannot retrieve loss of access time within the same time range. Time intervals may be reduced to increase the number of time ranges but this would increase the computation time	**Limited by cell size** According to Nelson [71], travel speed on tracks for motorized vehicles is 10 km/h and walking speed on bare ground is 2.5 km/h. Considering the granularity of the cell the time resolution would be 6 min for motorized vehicles and 24 min for walking
Computation time Calculated for the specific analysis of Cyclone Idai case study with the computational environment described in Additional file 3	** ~ 3.5 h** The main time consuming step was the isochrones processing	** ~ 1 h** The main time consuming step was downloading the friction layer

The main limitation of the network-based method was the completeness of the road network. All areas not reached by a road in the network model will be considered out of range, while the raster-based method will consider open space routing. This limitation explains the difference between the two approaches (see Additional file 2: Fig. S4). The network-based estimation of the number of inhabitants living beyond a walking distance of six hours to the nearest health facility is presumably overestimated compared to the raster-based approach. Therefore, in areas with low network density—specifically if the network is not sufficiently mapped—the network-based approach will tend to overestimate the time required to access health facilities. Other more complex network methods like two-step floating catchment would suffer the same trade-offs in data quality. In such contexts, the use of a raster-based approach with a friction layer from the Malaria Atlas Project may provide more accurate results. A potential solution is the fusion of raster layers provided by both approaches—e.g. by picking the lower value of estimated travel time.

On the other hand, the network-based method does not suffer from the fixed raster resolution (see Additional file 2: Fig. S5). In areas with high levels of completeness and accuracy of the OSM network, the network-based approach can be considered more accurate. As OSM data is updated continuously it can be expected to increase in completeness and accuracy and to also reflect new road construction work in a timely manner. The friction layer used by the raster-based approach is not automatically updated. As of September 2021, there has been only one release for the walking friction layer and a 5 year update interval for the motorized friction layer. For future research, our work provides insight into the creation of a workflow to update friction layers with the latest available main input datasets: road network, land cover, and elevation.

The assumptions of the network-based approach need to be discussed critically: We analyzed walking and driving as separate means of transport. However, it has to be assumed that multimodal transportation (walking and moto-taxi) is not uncommon in the case study region. Therefore, our method is likely to overestimate or underestimate the impacted population respectively for walking-only and driving profiles. The development of tools for a multimodal analysis could improve the precision of the results. Technically, the weighted graph used by openrouteservice builds on the type of highway registered in the OSM dataset. The walking-only profile considers any road class with the avoidance of unsafe highways. Therefore, we recommend the introduction of a new profile considering motorized vehicles on highways which allow driving and walking otherwise. The motorized friction layer used in the raster-based method provides a basic estimation of multimodal transport as it assigns an adjusted walking speed on open spaces without road access. Nevertheless, an adequate multimodal analysis would require prior knowledge of the modal behavior of the study area. Such information was not available for Mozambique and is generally difficult to acquire for large parts of the Global South, specifically for rural areas.

Our work also provides an insight for future analysis on the accessibility to healthcare facilities for other kinds of natural and man-made disasters with the prerequisite of the availability of disaster datasets.

Our case study demonstrates the feasibility of an accessibility analysis workflow in identifying the vulnerability of populations to the loss of accessibility to healthcare after a disaster of the magnitude of Cyclone Idai. We showed the potential support that such analysis could provide to operational decision making by governmental agencies and humanitarian actors:

- The rapid identification of populations experiencing a loss in accessibility to healthcare could help operational actors with deciding where to focus their interventions.
- In disaster contexts, where actors are not familiar with the area and information on population distribution is not easily available, our workflow enables rapid identification of population nuclei and mitigates the risk of overlooking areas due to a lack of information. Therefore, it could be integrated within a needs assessment protocol.
- The analysis also allows for identification of indirectly impacted areas and inhabitants that may not have been identified otherwise. As even if the floods did not occur in these areas, they impact access to healthcare facilities.
- Moreover, these analyses may be used for preparedness plans by providing a simulation tool to assess the potential impact of future disasters. The extent of historic floods or the extent of flood risks could then be used as input for the disaster context situation.
- Also, these analyses are relevant to be used to identify the remoteness of populations and their vulnerability to accessing healthcare regardless of the circumstances.

### Road criticality analysis

Our centrality analysis showed the relevancy of the targeted edge betweenness centrality indicator in assessing critical road segments in a network for populational access to healthcare facilities. This indicator mitigates the limitations of more classic centrality indicators:

- The destination of the road network can easily be defined to a list of points such as healthcare facilities. We can also imagine other relevant use cases such as tsunami or earthquake evacuation roads.
- By default, we used the 1 km^2^ aggregated population nuclei as origins for the road networks. These points can be modified to adjust the resolution of population nuclei or to set other origin points.
- Finally, as the origin and destination points can be adjusted and are fewer than the network’s nodes, the computation time is reduced and enabled to widen the area of interest.

As it enables the identification of road segments prone to blockage due to flooding as well as the main backup roads in such a situation, this analysis can support decision making for disaster preparedness planning or defining evacuation roads.

However, the flood mask is not free of uncertainty due to its spatial resolution and the update frequency. The flood mask indicates presence of water but cannot tell if a road is passable. For instance, shallow flooding might be passable at slow speeds. Frequent updates of the road status, e.g. based on feedback by drivers or volunteers, could resolve this issue. Another critical point of our set-up is the intersection of the road network with the flood layer. Currently, a road segment is considered flooded if one of the nodes intersects the flood mask. If the road segment in between two nodes intersects the flood mask but the two nodes do not, the road segment would be considered as not flooded. This could lead to errors, especially if the road segment has a high centrality score.

Also, the centrality analysis does not provide any information regarding the capacity of the road segments to face an increase of traffic. Hence, it is not possible to consider potential traffic congestion. However, the approach highlights roads that are critical for access to health sites given the effects of a potential disaster event. These roads qualify for a real world assessment of their capacity. Potential bottlenecks could thereby be identified and prioritized for upgrading by national and international actors.

During emergency responses, one may argue that any intervention should be maximizing the number of people reached. If such a strategy is pursued, the targeted centrality could be adapted to a population-weighted indicator. This indicator would give higher importance to departure points with larger populations. Therefore, it must be handled carefully as it presents an inequitable result. Remote population nuclei, who are likely to already face lower accessibility to healthcare, risk becoming invisible. Therefore, this population-weighted indicator should not be used by default but should be justified by operational decisions.

## Conclusion and outlook

Organizing the different phases of the disaster management cycle and its consequences toward accessibility to health facilities requires information on the vulnerabilities of populations and critical infrastructure. In many regions of the Global South, available data is often scarce and contradicts with the reactivity required by emergency responses or with the available resources to define preparedness and mitigation plans. We have demonstrated that the use of geospatial analysis and open access data can help to resolve these challenges. In regions where the OSM road network is incomplete, the raster-based approach built on open data such as the MAP layer should be preferred. If the OSM road network is sufficiently complete, the network-based approach offers a higher spatial resolution for road-based modes of transport. As open space routing is not represented in the network-based approach, it should be combined with the results of the raster-based approach. For areas with sufficient completeness of the OSM road network the t*argeted edge betweenness centrality* indicator provides important information to assess the criticality of the road in providing physical access to health facilities. Furthermore, it allows the identification of the most critical and the main backup roads.

Further work is needed to test the applicability of the targeted edge centrality centrality indicator in the context of different disasters and geographical contexts. Furthermore, we encourage future research to focus on the construction of a multimodal network-based approach and the combination of raster- and network-based methods to overcome their respective limitations.

In addition, future work is encouraged towards the automatization of network and raster analysis. From our perspective, one of the more important next steps is the management, editing, and sharing of flood extents. Further, automatization of the workflow is essential for the testing of these approaches with relevant humanitarian actors.

## Supplementary Information

**Additional file 1: **Data availability and completeness. **Fig. S1.** Indicator comparing population building densities from Ohsome Quality Analyst to evaluate the completeness of OSM data. **Fig. S2.** Comparison of mapped health facilities in OSM with field observation: facilities inventory, localisation comparison and damages inventory.

**Additional file 2: Fig. S3. **Accessibility workflow: raster- and network-based methods. **Fig. S4.** Accessibility to health facility, raster- vs. network-based method comparison A. Under six hours walking time to access healthcare estimations between raster- and network-based methods and B. mapped infrastructures. **Fig. S5** Accessibility to health facilities in urban context, raster- vs. network-based method comparison A. population estimation B. extent of under 6 walking hours access to health facilities. **Table S1.** Comparison of mapped health facilities in OSM with field observation: facilities inventory, localisation comparison and damages inventory.

**Additional file 3: **Performance and computational environment.

## Data Availability

The dataset(s) supporting the conclusions of this article are available through the scripts in the GitHub repository, https://github.com/GIScience/healthcare-access-idai: Project name: Healthcare accessibility and road criticality during floods disaster. Programming language: R. Other requirements: GDAL 3.2.1, PROJ 7.2.1 and GEOS 3.9 or later
