# Supplementary material for: Assessing road criticality and loss of healthcare accessibility during floods: the case of Cyclone Idai, Mozambique 2019
Source: Int J Health Geogr. 2022 Oct 12;21:14. doi: 10.1186/s12942-022-00315-2 (PMC9559768; doi:10.1186/s12942-022-00315-2)
Supplement: Supplementary file 1 — Additional file 1: Data availability and completeness. Fig. S1. Indicator comparing population building densities from Ohsome Quality Analyst to evaluate the completeness of OSM data. Fig. S2. Comparison of mapped health facilities in OSM with field observation: facilities inventory, localisation comparison and damages inventory. [file 12942_2022_315_MOESM1_ESM.pdf]

Additional file 1: Data availability and completeness

We used another indicator to estimate the completeness of OSM features mapped through remote mapping campaigns such as roads and buildings (1). We compared the density of these features in OSM and population density from an external dataset (worldpop) and used it as an estimation of feature completeness. The correlation between roads and population density is not straightforward, thus we used this indicator on building completeness as a proxy for road completeness. High scores of the indicators imply that feature density is well aligned with population density which was taken as an indication of a relatively high completeness. On the contrary, a low score was interpreted as an indication that many features were not mapped and thus taken as an indication for a low feature completeness. The indicator was calculated for the whole country using a regular tessellation of 346 hexagons. Each hexagon covered 2903 km^2. We used the Ohsome Quality Analyst (2) to calculate the indicator, taking a snapshot of the OSM database from the 20th of September 2021. The population and building comparison indicator (fig. S1) indicated higher scores in the center of the country. This coincides with our study area and in particular with the areas affected by the floods. This indicates a higher building completeness in the areas affected by the floods than in the rest of the country at the time of the analysis in September 2021, which most likely indicates the same for roads.

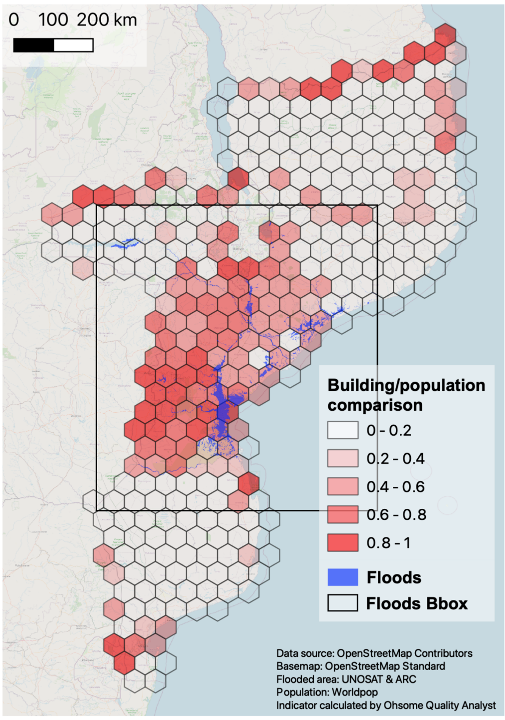

Figure S1. Indicator comparing population building densities from Ohsome Quality Analyst to evaluate the completeness of OSM data.

Furthermore, we interviewed a humanitarian field worker who participated in the emergency response in the wake of the cyclone in the area of Dondo, Sofala Province. Both his observations about health facilities and the GPS coordinates collected by him were used as auxiliary data to judge the quality of the OSM data. The field worker visited seven health facilities in the Dondo District which were part of the study region. His information (Figure S2) allowed us to assess whether all the health facilities were mapped in OSM in the sample region and if their location was accurate. A lack of location precision would affect the analysis as it focused on the accessibility and the criticality of the roads to access these sites. Of the seven facilities visited, two health centers (Savane CS and Thundane CS) were not mapped in OSM. Moreover, the location of several health facilities in OSM (Chibuabuabua CS, Mutua CS, and Mafambisse CS) lacked spatial precision. They were mapped to within several hundred meters of the actual locations. Finally, in the same area, three facilities were mapped in OSM which were unknown to the field worker.

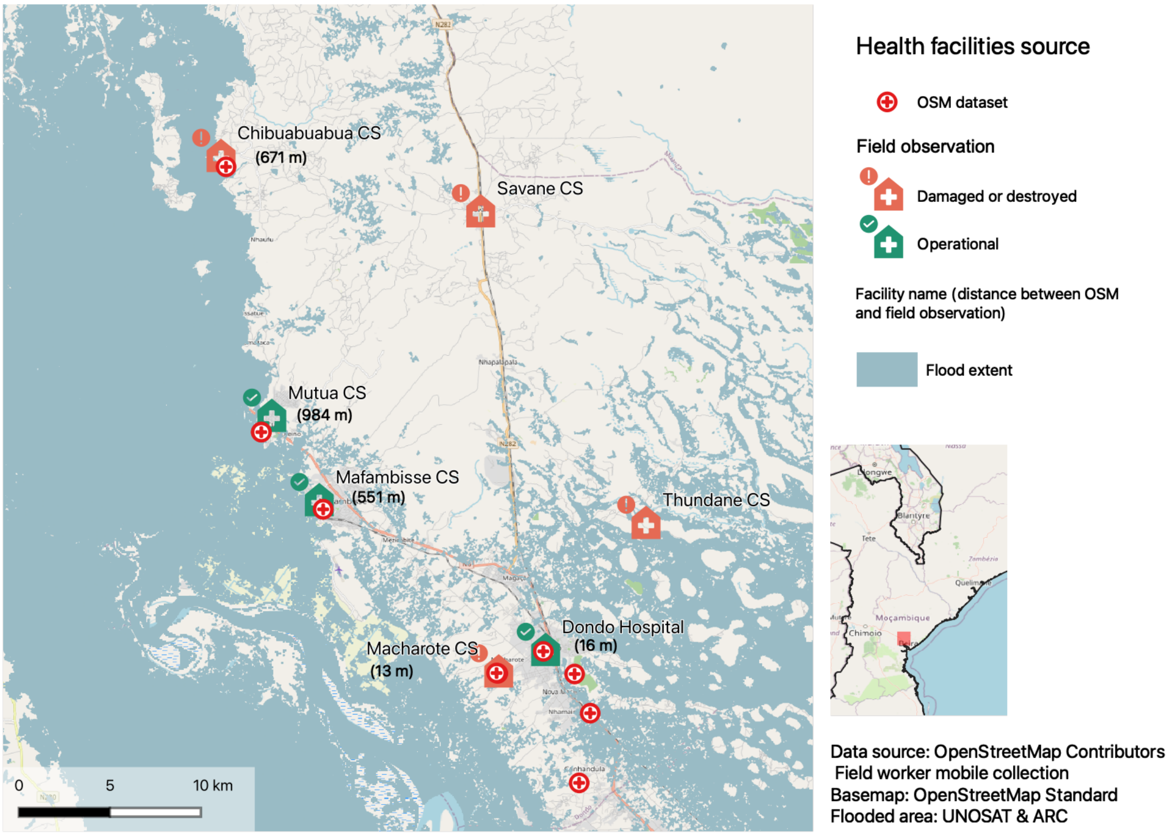

Figure S2. Comparison of mapped health facilities in OSM with field observation : facilities inventory, localisation comparison and damages inventory.

References:

1. Scholz S, Knight P, Eckle M, Marx S, Zipf A. Volunteered Geographic Information for Disaster Risk Reduction—The Missing Maps Approach and Its Potential within the Red Cross and Red Crescent Movement. Remote Sens. 2018 Aug;10(8):1239.

2. ohsome quality analyst [Internet]. [cited 2021 Oct 25]. Available from: https://oqt.ohsome.org/index.html
