# Supplementary material for: Assessing road criticality and loss of healthcare accessibility during floods: the case of Cyclone Idai, Mozambique 2019
Source: Int J Health Geogr. 2022 Oct 12;21:14. doi: 10.1186/s12942-022-00315-2 (PMC9559768; doi:10.1186/s12942-022-00315-2)
Supplement: Supplementary file 2 — Additional file 2: Fig. S3. Accessibility workflow: raster- and network-based methods. Fig. S4. Accessibility to health facility, raster- vs. network-based method comparison A. Under six hours walking time to access healthcare estimations between raster- and network-based methods and B. mapped infrastructures. Fig. S5 Accessibility to health facilities in urban context, raster- vs. network-based method comparison A. population estimation B. extent of under 6 walking hours access to health facilities. Table S1. Comparison of mapped health facilities in OSM with field observation: facilities inventory, localisation comparison and damages inventory. [file 12942_2022_315_MOESM2_ESM.pdf]

Additional file 2: Supplementary figures and tables

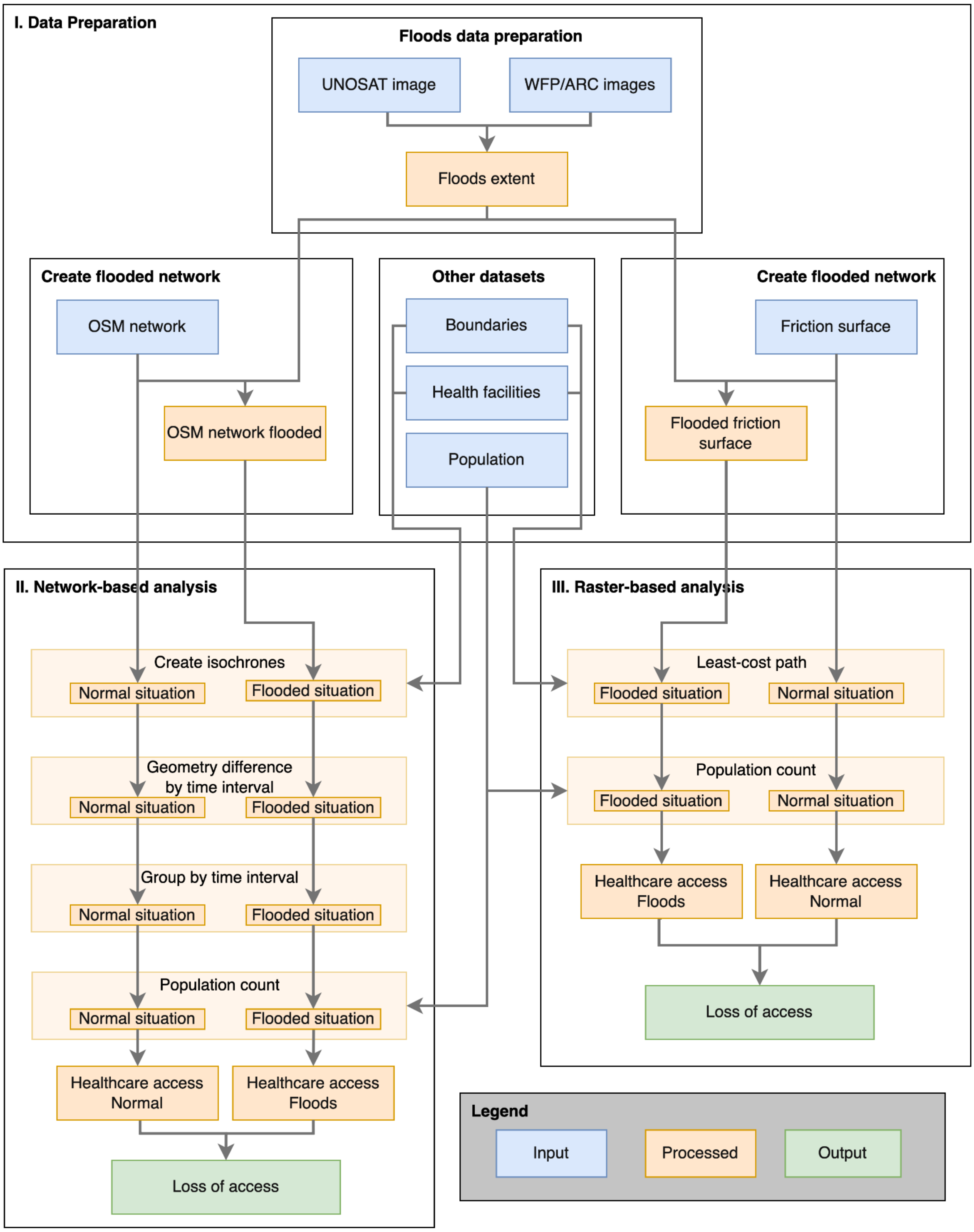

Figure S1. Accessibility workflow: raster- and network-based methods

Package	Reference	Package	Reference
ggpubr	Alboukadel Kassambara (2020). ggpubr: 'ggplot2' Based Publication Ready Plots. R package version 0.4.0. https://CRAN.R-project.org/package=ggpubr	ggplot2	H. Wickham. ggplot2: Elegant Graphics for Data Analysis. Springer-Verlag New York, 2016.
geojsonsf	David Cooley (2020). geojsonsf: GeoJSON to Simple Feature Converter. R package version 2.0.1. https://CRAN.R-project.org/package=geojsonsf	knitr	Yihui Xie (2021). knitr: A General-Purpose Package for Dynamic Report Generation in R. R package version 1.33.
units	Pebesma E, Mailund T, Hiebert J (2016). “Measurement Units in R.” _R Journal_, *8*(2), 486-494. doi: 10.32614/RJ-2016-061 (URL: https://doi.org/10.32614/RJ-2016-061).	gridExtra	Baptiste Auguie (2017). gridExtra: Miscellaneous Functions for "Grid" Graphics. R package version 2.3. https://CRAN.R-project.org/package=gridExtra
RJSONIO	Duncan Temple Lang and Jonathan Wallace (2020). RJSONIO: Serialize R Objects to JSON, JavaScript Object Notation. R package version 1.3-1.4. https://CRAN.R-project.org/package=RJSONIO	grid	R Core Team (2021). R: A language and environment for statistical computing. R Foundation for Statistical Computing, Vienna, Austria. URL https://www.R-project.org/.
geojsonio	Scott Chamberlain and Andy Teucher (2021). geojsonio: Convert Data from and to 'GeoJSON' or 'TopoJSON'. R package version 0.9.4. https://CRAN.R-project.org/package=geojsonio	OpenStreet Map	Ian Fellows and using the JMapViewer library by Jan Peter Stotz (2019). OpenStreetMap: Access to Open Street Map Raster Images. R package version 0.3.4. https://CRAN.R-project.org/package=OpenStreetMap
RCurl	Duncan Temple Lang (2021). RCurl: General Network (HTTP/FTP/...) Client Interface for R. R package version 1.98-1.3. https://CRAN.R-project.org/package=RCurl	tmaptools	Martijn Tennekes (2021). tmaptools: Thematic Map Tools. R package version 3.1-1. https://CRAN.R-project.org/package=tmaptools
sfnetworks	Lucas van der Meer, Lorena Abad, Andrea Gilardi and Robin Lovelace (2021). sfnetworks: Tidy Geospatial Networks. R package version 0.5.2. https://CRAN.R-project.org/package=sfnetworks	tmap	Tennekes M (2018). “tmap: Thematic Maps in R.” _Journal of Statistical Software_, *84*(6), 1-39. doi: 10.18637/jss.v084.i06 (URL: https://doi.org/10.18637/jss.v084.i06).
tidygraph	Thomas Lin Pedersen (2020). tidygraph: A Tidy API for Graph Manipulation. R package version 1.2.0. https://CRAN.R-project.org/package=tidygraph	polylabelr	Larsson J (2020). _polylabelr: find the pole of inaccessibility (visual center) of a polygon_. R package version 0.2.0, <URL: https://cran.r-project.org/package=polylabelr>.

classInt	Roger Bivand (2020). classInt: Choose Univariate Class Intervals. R package version 0.4-3. https://CRAN.R-project.org/package=classInt	exactextractr	Daniel Baston (2021). exactextractr: Fast Extraction from Raster Datasets using Polygons. R package version 0.6.0. https://CRAN.R-project.org/package=exactextractr
jsonlite	Jeroen Ooms (2014). The jsonlite Package: A Practical and Consistent Mapping Between JSON Data and R Objects. arXiv:1403.2805 [stat.CO] URL https://arxiv.org/abs/1403.2805.	rjson	Alex Couture-Beil (2018). rjson: JSON for R. R package version 0.2.20. https://CRAN.R-project.org/package=rjson
httr	Hadley Wickham (2020). httr: Tools for Working with URLs and HTTP. R package version 1.4.2. https://CRAN.R-project.org/package=httr	sfheaders	David Cooley (2020). sfheaders: Converts Between R Objects and Simple Feature Objects. R package version 0.4.0. https://CRAN.R-project.org/package=sfheaders
gdistance	van Etten, J. (2017). R package gdistance: Distances and routes on geographical grids. Journal of Statistical Software, 76(1), 1–21. https://doi.org/10.18637/jss.v076.i13	rmapshaper	Andy Teucher and Kenton Russell (2020). rmapshaper: Client for 'mapshaper' for 'Geospatial' Operations. R package version 0.4.4. https://CRAN.R-project.org/package=rmapshaper
doParallel	Microsoft Corporation and Steve Weston (2020). doParallel: Foreach Parallel Adaptor for the 'parallel' Package. R package version 1.0.16. https://CRAN.R-project.org/package=doParallel	stars	Edzer Pebesma (2021). stars: Spatiotemporal Arrays, Raster and Vector Data Cubes. R package version 0.5-2. https://CRAN.R-project.org/package=stars
osmextract	Andrea Gilardi and Robin Lovelace (2021). osmextract: Download and Read OpenStreetMap Data Extracts. R package version 0.2.1. https://CRAN.R-project.org/package=osmextract	tictoc	Sergei Izrailev (2021). tictoc: Functions for Timing R Scripts, as Well as Implementations of Stack and List Structures. R package version 1.0.1. https://CRAN.R-project.org/package=tictoc
tidyverse	Wickham et al., (2019). Welcome to the tidyverse. Journal of Open Source Software, 4(43), 1686, https://doi.org/10.21105/joss.01686	raster	Robert J. Hijmans (2020). raster: Geographic Data Analysis and Modeling. R package version 3.4-5. https://CRAN.R-project.org/package=raster
sf	Pebesma, E., 2018. Simple Features for R: Standardized Support for Spatial Vector Data. The R Journal 10 (1), 439-446,	readxl	Hadley Wickham and Jennifer Bryan (2019). readxl: Read Excel Files. R package version 1.3.1. https://CRAN.R-project.org/package=readxl

	https://doi.org/10.32614/RJ-2018-009		
rgeoboundaries)			

Table S1. Comparison of mapped health facilities in OSM with field observation : facilities inventory, localisation comparison and damages inventory.

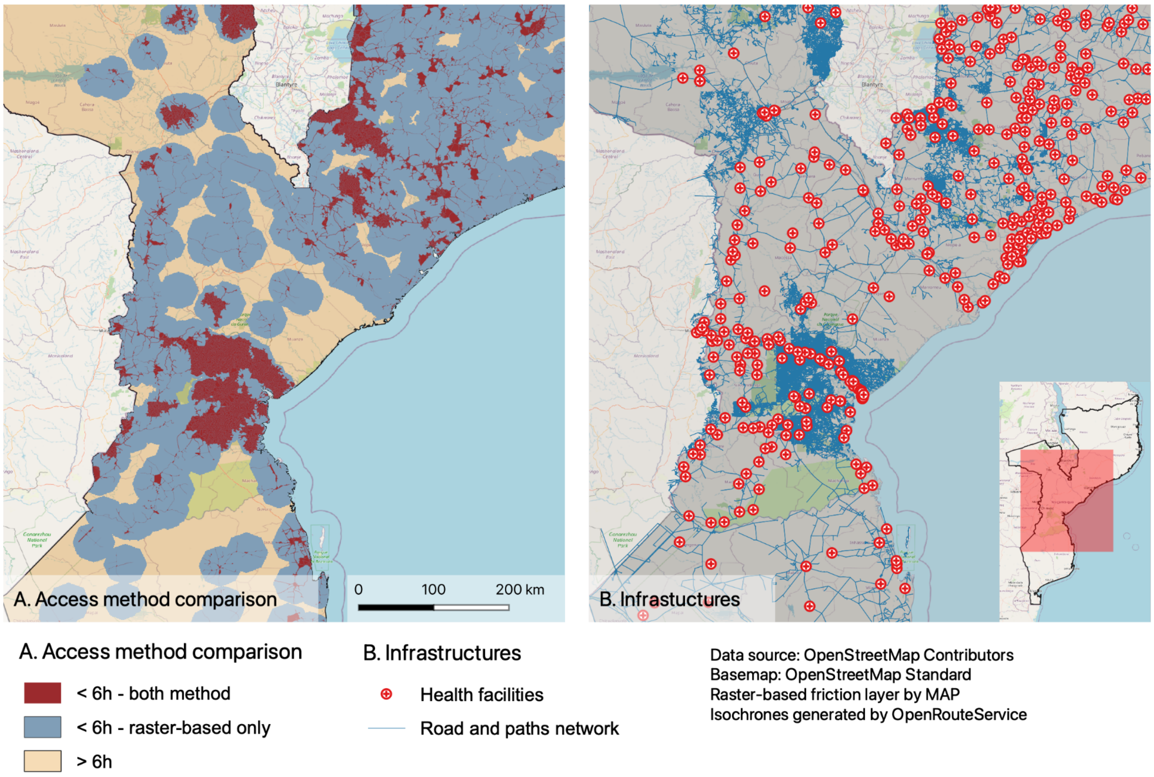

Figure S2. Accessibility to health facility, raster- vs. network-based method comparison A. Under six hours walking time to access healthcare estimations between raster- and network-based methods and B. mapped infrastructures

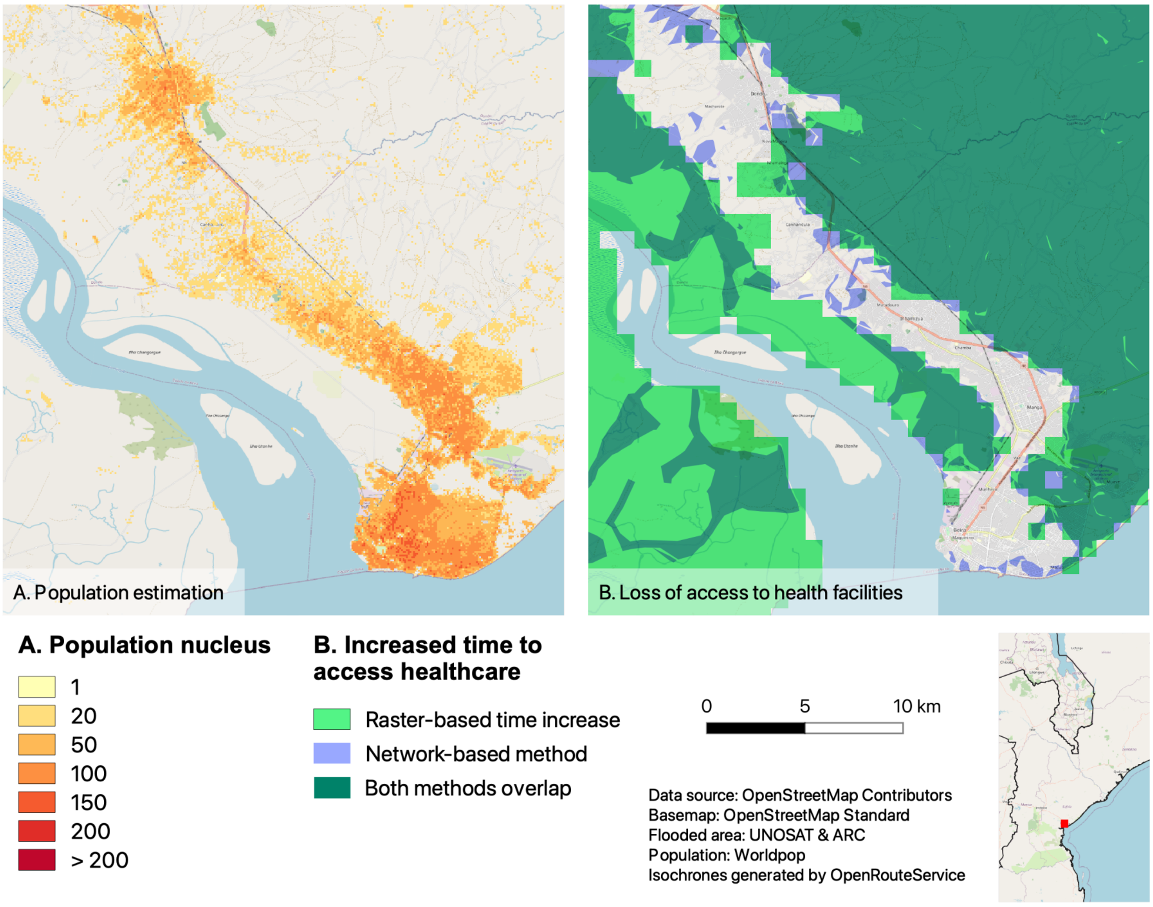

Figure S3. Accessibility to health facilities in urban context, raster- vs. network-based method comparison A. population estimation B. extent of under 6 walking hours access to health facilities
