# Supplementary material for: Assessing road criticality and loss of healthcare accessibility during floods: the case of Cyclone Idai, Mozambique 2019
Source: Int J Health Geogr. 2022 Oct 12;21:14. doi: 10.1186/s12942-022-00315-2 (PMC9559768; doi:10.1186/s12942-022-00315-2)
Supplement: Supplementary file 3 — Additional file 3: Performance and computational environment. [file 12942_2022_315_MOESM3_ESM.pdf]

# Additional file 3 : Performance and computational environment

## Parameters of the estimation

We extracted the running time in order to give an estimation.

Scope of the estimation:

- Country: Mozambique
- Profile : foot-walking
- Health care level OSM tags :

| OSM tags | All |
|---|---|
| **amenity** | clinic, health_post, doctors, hospital |
| **healthcare** | clinic, health_post, doctors, midwife, nurse, center, hospital |
| **building** | hospital |
| **Total points** | 1060 |

## Analysis steps

### I. Data Preparation

| | Tech | Duration (min) |
|---|---|---|
| **Total** | | **143** |
| 1. Data download | | 28 |
| *1.1 Main datasets* | *R script* | *4* |
| *1.2 Preparation of the flood data* | *R script* | *24* |
| 2. Create a .osm.pbf file | R script | 115 |

### II. Healthcare accessibility analysis

| Vector-based access analysis | Tech | Driving-car Duration (min) | Foot-walking Duration (min) |
|---|---|---|---|
| **Total** | | **60** | **55** |
| 1. Launch ORS instances | Docker | 19 | 19 |
| 2. Isochrone processing | R script | 41 | 36 |

Note: The process depends on the data preparation. Therefore to get a total processing time, you should take into consideration both process steps.

| Raster-based access analysis | Tech | Multimodal car Duration (min) | Foot-walking Duration (min) |
|---|---|---|---|
| **Total** | R script | **34** | **20** |

Note: The process depends only on the first section of the data preparation (I.1. Data download) of the data preparation and does not require the second section (I.2. Create a .os.pbf file). Therefore to get a total processing time, you should take into consideration the data download steps.

### III. Centrality analysis

| | Tech | Foot-walking Duration (h) |
|---|---|---|
| **Total** | | ~ 60 |
| 1. Launch ORS instances | Docker | 0.3 |
| 2. Targeted centrality | R script | ~ 60 |

Note: the processing time was calculated for the Bbox corresponding to the whole Mozambique. The processing time is highly dependent on the chosen bounding box and a reduced area of interest would reduce the computation time.

## Computational environment

This estimation was done with the following system:

| | |
|---|---|
| OS | MacOS 11.3 |
| RAM | 16 Go |
| R | 4.0.5 |
| GDAL | 3.3.0 |
| GEOS | 3.9.1 |
| PROJ | 8.0.1 |
